# Detection of Pathogenic Microbe Composition Using Next-Generation Sequencing Data

**DOI:** 10.3389/fgene.2020.603093

**Published:** 2020-11-30

**Authors:** Haiyong Zhao, Shuang Wang, Xiguo Yuan

**Affiliations:** ^1^School of Computer Science and Technology, Liaocheng University, Liaocheng, China; ^2^School of Computer Science and Technology, Xidian University, Xi’an, China

**Keywords:** microbe composition detection, microbe abundance estimation, next-generation sequencing, 16S rRNA, machine learning

## Abstract

Next-generation sequencing (NGS) technologies have provided great opportunities to analyze pathogenic microbes with high-resolution data. The main goal is to accurately detect microbial composition and abundances in a sample. However, high similarity among sequences from different species and the existence of sequencing errors pose various challenges. Numerous methods have been developed for quantifying microbial composition and abundance, but they are not versatile enough for the analysis of samples with mixtures of noise. In this paper, we propose a new computational method, PGMicroD, for the detection of pathogenic microbial composition in a sample using NGS data. The method first filters the potentially mistakenly mapped reads and extracts multiple species-related features from the sequencing reads of 16S rRNA. Then it trains an Support Vector Machine classifier to predict the microbial composition. Finally, it groups all multiple-mapped sequencing reads into the references of the predicted species to estimate the abundance for each kind of species. The performance of PGMicroD is evaluated based on both simulation and real sequencing data and is compared with several existing methods. The results demonstrate that our proposed method achieves superior performance. The software package of PGMicroD is available at https://github.com/BDanalysis/PGMicroD.

## Introduction

In the last decade, metagenomics has emerged as a remarkable event in the study of microbial ecology (Lindner and Renard, 2013). The detection of pathogenic microbial composition (i.e., species and their abundance) is very important in this field since it can provide valuable information for supporting pathogenic treatment and in the fields of ecology and human health (Chaudhary et al., 2015). Next-generation sequencing (NGS) provides an unprecedented opportunity to explore the composition of microbes in a sample (Fuhrman, 2012). 16S rRNA from microbes contains regions of highly conserved and highly variable sequences, providing a reliable substrate for species identification (Teeling and Glockner, 2012). Currently, a set of methods have been developed to analyze microbial composition using 16S data. One early approach, BLAST (Scott and Madden, 2004),calculates the similarity score for local alignments of reads against reference sequences to explore the microbial diversity of complex environments.

Recently, similarity-based clustering algorithms (Kopylova et al., 2012; Mahe et al., 2014; Albanese et al., 2015; Al-Ghalith et al., 2016) have superseded the traditional technologies. For example, Mothur (Yin et al., 2014) detects the species in samples via clustering the whole sequencing reads into an operational taxonomic unit (OTU) and then selects the representative read to map to the reference library. QIIME (Caporaso et al., 2010) uses open reference OTU picking for statistical analysis of clusters, abundances, and taxonomy. Open-reference OTU picking uses a database of known 16S genes to create OTU clusters while it allows for the formation of OTUs, which have sequences sufficiently different from the references. Phylogenetic techniques, such as neighbor-joining (Price et al., 2009) and Bayesian posterior probability (Matsen et al., 2010), have emerged, which can make specific taxonomic assignments but within a large computational burden (Bazinet and Cummings, 2012). Also, Karp (Reppell and Novembre, 2018) and Kallisto (Bray et al., 2016) used a k-mer based pseudo alignment approach to classify 16S rRNA reads using a reference library of known species and then deduced the types of species and their abundance according to the classified reads. One of the key points is to derive a reliable mapping result of the reads to the references. However, high similarity among species sequences and sequencing errors make it extremely difficult. Harp (Kessner et al., 2013) analyzed microbial composition by estimating haplotype frequencies based on an expectation-maximization algorithm. Such a method allows for mapping uncertainty in the reads but relies on a probability model. No available methods are powerful enough to fully explore microbial composition in the case of highly mixed species in a sample.

In this paper, we propose a new computational tool, PGMicroD, to detect pathogenic species and their abundance using 16S rRNA. After filtering the potentially mistakenly mapped reads, it extracts multiple species-related features based on a deep exploration of the statuses of read alignment and then trains an Support Vector Machine (SVM) classifier to predict types of species. Subsequently, all the multiple-mapped reads from 16S are re-mapped to the references of the predicted species, and the relative abundance of each species is estimated by counting the mapped reads. We test the performance of PGMicroD based on both simulation and real sequencing data, and we show that the new proposed tool outperforms several existing methods. Machine learning has a wide range of applications in the field of pattern recognition, especially for classification problems, because of high speed and high prediction accuracy. We start from the perspective of a classification algorithm, fully considering high similarity among species, sequencing errors, and the status of read distributions, to achieve more accurate species detection.

In section “Materials and Methods,” we demonstrate the implementation of the PGMiroD method, including the primary theorem and method implementation. In section “Results and Discussion,” we explore three major factors associated with the performance of detecting pathogenic species and describe experiments to test PGMicroD on simulation and real datasets. Finally, the conclusions are summarized in section “Discussion and Conclusion.”

## Materials and Methods

### Workflow of PGMicroD

**FIGURE 1:**
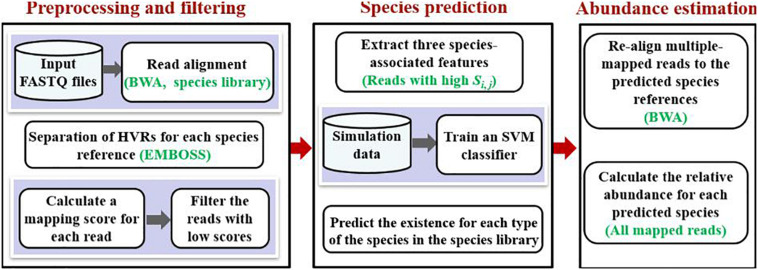
The framework of the PGMicroD method for predicting pathogenic microbial species and their abundances by using NGS data.

The workflow chart of PGMicroD is depicted in Figure 1. It consists of three primary steps: preprocessing and filtering, species prediction, and abundance estimation.

(1) *Preprocessing and filtering*: The input data is a FASTQ file from the sample to be analyzed and is aligned to the reference library via the classic algorithm BWA (Li and Durbin, 2009). The unsuccessfully aligned reads are removed from the FASTQ file. Seven hypervariable regions (HVRs) (V2–V8) in 16S rRNA are separated from each of the reference library via the EMBOSS Tool (Mullan and Bleasby, 2002). For each aligned read, we propose a new mapping score to express the confidence of one read originating from a specific reference. Then, the aligned reads with low scores are filtered from the alignment result.

(2) *Species prediction*: After filtering the mapped reads with low mapping scores, the remainder of the mapped reads are used to predict species via further analysis. For one species reference, we extract three types of signatures as features (which have implications for composition detection) to feed into the trained SVM classifier to determine whether this species exists in the sample or not.

(3) *Abundance estimation*: With the detected species, the filtered reads that aligned to multiple references (multiple-mapped reads) are re-aligned to the corresponding references. Compared to the first-round alignment, where all the references in the library are used and false species references have a great interference on the mapping results, the alignment on the detected species references is more reasonable. Finally, we use the mapped reads to estimate the relative abundance of each species.

We provide further details on the principles underlying this work and the implementation of each of the steps.

### Preprocessing and Filtering

**FIGURE 2:**
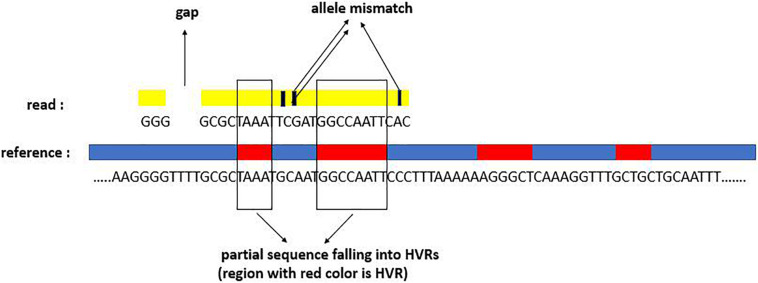
Three alignment signatures of one read aligned to one reference: mapping gap, allele mismatch, and subsequences falling into HVRs.

For the successfully mapped reads (i.e., they are successfully mapped to at least one reference in the reference database), we carry out further analysis for the alignment status. Generally, one mapped read can display three types of signatures: mapping quality, mapping gaps, and mapped subsequences overlapped with the HVRs. For a clear understanding of the mapping signatures, we depict a mapping example in Figure 2. The example shows that there is one gap, three allele mismatches, and two subsequences covering two HVRs.

Mapping quality, which can be observed from the SAM file of the alignment result, displays the mapping confidence of each position for one read, including the match and mismatch. Due to the co-existence of sequencing errors, disturbance of other unknown species, and high similarity between pathogenic species, there are several multiple-mapped reads. Theoretically, each read should have originated from one species. The phenomenon of multiple mapping could have several major characteristics: (1) the reads are consistent across multiple species and are thus mapped to the corresponding references with high confidence; (2) the reads are mapped to the extremely similar references; (3) for one read mapped to multiple references, the mapping confidence might be different, i.e., some of them might have low confidence. With such considerations, we make a further analysis of the mapped reads to improve mapping effectiveness. We start from each reference mapped by at least one read and then determine if each mapped read should be retained or discarded from the reference. For one read, if it owns higher mapping quality, a smaller amount of gap, and a larger area of subsequence overlapped with HVRs, then its mapping result has more confidence.

Therefore, we define three indices for measuring above three signatures. For convenient description, we assume the reference set as *Z* = {*z*_1_, *z*_2_, …, *z*_*M*_} with the total number of references *M*, and the sequencing read set as *R* = {*r*_1_, *r*_2_, …, *r*_*N*_} with the total number of reads *N*. For one read *r*_*i*_ with *i*∈1, …, *N* and length *L*_*i*_, let (*r*_*i*_[1], *r*_*i*_[2], …, *r*_*i*_[*L*_*i*_]) be the base call vector at each position along the read, and (*Q*_*i*_[1], *Q*_*i*_[2], …, *Q*_*i*_[*L*_*i*_]) be the base quality score vector of this read. For reference sequence z*_*j*_* with *j*∈1, …, *M* aligned by the read *r*_*i*_, let (*z*_*j,i*_[1], *z*_*j,i*_[2], …, *z*_*j,i*_[*L*_*i*_]) give the base calls vector of reference *z*_*j*_ corresponding to every position of the read. Note the entries in this vector may not be contiguous due to insertions, deletions, or because the reads are paired-end. With the mapping quality, we could calculate the corresponding probability for the sequencing error vector, (*q*_*i*_[1], *q*_*i*_[2], …, *q*_*i*_[*L*_*i*_]), by using the expression of *Q*_*i*_[*k*] = −10^∗^lg(*q*_*i*_[*k*]) with *k*∈1, 2, …, *L*_*i*_. Accordingly, the confidence (*P*_*i*_, *_*j*_*) of the read *r*_*i*_ mapped to the reference *z*_*j*_ could be expressed as:

$$p_{i,j}=\prod_{k=1}^{L_{i}}p\,\left(r_{i}\,\left[k\right]\right),\mathrm{where}$$

(1) $$p\,\left(r_{i}\,\left[k\right]\right)=\left\{ \begin{array}{cc} 1-q_{i}\,\left[k\right], & r_{i}\,\left[k\right]=z_{j,i}\,\left[k\right]\\ q_{i}\,\left[k\right]/3, & r_{j}\,\left[k\right]\neq z_{j,i}\,\left[k\right] \end{array}\right..$$

In the formulation (1), because each base is independent of another, we use (*P*_*i*_, *_*j*_*) as a joint probability of all bases. It is noted that (*P*_*i*_, *_*j*_*) is a positive value always less than 1.

As far as the mapping gap is concerned, the phenomenon of insertion, deletion, soft mapping, and hard mapping is regarded as a “gap.” If the read *r*_*i*_ is mapped to the reference *z*_*j*_, the mapping gap is quantified as the log2 conversion of the summation of gaps, which is expressed as:

(2) $$R_{g\,a\,p}=\log_{2}\left(\sum_{k=1}^{K}x_{k}+1\right).$$

where *x*_*k*_ is the width of the *k*-th gap and *K* is the number of gaps in the read. In formulation (2), *x*_*k*_ means the width of the *k*-th gap, which is greater than or equal to 0. The sum of the whole gap is a positive integer greater than 0. By taking the log, it can be transformed to [0,1]. It is noted that when there is no gap in the read, the log operation makes no sense. Hence, we add a constant value 1 into the formulation. If exponential operation is used in *x*_*k*_, the result will be more than 1. The approach cannot keep the quantization of gap consistent with the dimension of (*P*_*i*_, *_*j*_*).

As for HVRs, it is well-known that the similarity of 16S rRNA on the species/strain level is up to 94%. The areas of HVRs display an important role for microbe composition identification since they are usually varying across species (whereas non-HVRs are usually consistent across species). Thus, the mapped read covering HVRs is significant for indicating a species reference. Accordingly, after *r*_*i*_ aligns to *z*_*j*_, the quantification of *r*_*i*_ for HVRs is expressed as Eq. 3.

(3) $$h_{i,j}=\frac{l\,e\,n\,g\,t\,h\,\,o\,f\,\,s\,q\,u\,e\,n\,c\,e\,\,o\,f\,\,r_{i}\,\,f\,a\,l\,l\,i\,n\,g\,\,i\,n\,t\,o\,\,H\,V\,R\,s\,\,o\,f\,\,z_{j}}{l\,e\,n\,g\,t\,h\,\,o\,f\,\,r_{i}}.$$

where *h*_*i,j*_ means the ratio of length of sequence in *i*-th read falling into HVRs of *j*-th reference after alignment. We combine the above three signatures and define a new mapping score (*S*_*i,j*_) for the read *r*_*i*_ mapped to the reference *z*_*j*_:

(4) $$S_{i,j}=\frac{P_{i,j}+h_{i,j}}{1+R_{g\,a\,p}}.$$

In formulation (4), (*P*_*i,j*_) and h_*i,j*_ are positively related to the possibility of species existence, but *R*_*gap*_ is negatively related to it. The above three values range in the same dimension; hence, we make them into ratio form. To avoid the case that the denominator is 0, we add the adjustment factor 1 in the denominator. The mapping score of each read can be computed via the formula (4) after alignment. If the score (*S*_*i,j*_) is less than a given threshold δ, then the read *r*_*i*_ is filtered from the alignment result. These reads with low mapping score are regarded as potential incorrect alignment status. We carry out a series of experiments to choose the appropriate value as δ and demonstrate the influence of the threshold value on the amount of correctly mapped reads.

### Species Prediction by Training an SVM Classifier

Species prediction (i.e., microbe composition detection) in a sample can be converted into a binary classification problem. Specifically, we extract multiple features associated with the existence of species in the sample, and establish a classifier to determine whether each species from the reference library exists in the sample or not. In the following text, we describe the implementation of the classifier and what features are extracted in detail.

#### Theorem of the SVM Classifier

We choose the SVM as the classifier for our problem, which is a state-of-the-art large margin classifier and gaining popularity in pattern recognition (Schuldt et al., 2004). The large-margin principle of SVM can avoid an over-fitting of classification. In addition, the kernel technique in SVM can resolve the linearly non-separable problem by making non-linear conversion of original data, which reflects distribution characteristics of the converted data in the extended space. Here, we provide a brief introduction to the theorem underlying SVM (Bennett and Campbell, 2000). The problem is to separate a set of data {(*x*_1_, *y*_1_), (*x*_2_, *y*_2_), …, (*x*_*n*_, *y*_*n*_)} into two classes, where *x*_*i*_ denotes a feature vector and *y*_*i*_ ∈ {−1, + 1} denotes its label. Generally, that two-class data can be separated by the hyperplane ***w***⋅***x***+ *b* = 0 in some specific space, where ***w*** is normal to the hyperplane and *b* controls the hyperplane to move parallel to itself. When we have no prior knowledge about the data distribution, then the optimal hyperplane is the one that can maximize the margin (Catoni, 2004). Generally, not all samples can be classified correctly. If the distance from the wrong-classified samples to the hyperplane is the smallest, one SVM classifier would be considered as an effective machine. Here, the relaxation factor in the SVM algorithm, *ξ*_*i*_, is used to measure the distance from one wrong-classified sample *x*_*i*_ to the hyperplane. The optimal values for *w* and *b* can be found by solving the constrained minimization problem shown in Eq. 5.

$$J=\min_{w}\left(\frac{1}{2}\,||w||+C\cdot \sum_{i=1}^{N}\xi_{i}\right)$$

(5) $$\;\,s.t.y_{i}\cdot \left(w^{T}\cdot x_{i}+b\right)\geq 1-\xi_{i}$$

$$\;\;\xi_{i}\geq 0\;\left(1\leq i\leq N\right).$$

The penalty parameter *C* is used to adjust the weight for the error of wrong-classified samples. The kernel technique (Bašić and Ribarić, 2002) in SVM can resolve the linearly non-separable problem by making non-linear conversion at original data, which reflects the distribution characteristics of the converted data in the extended space. Here, the RBF (radial basis function) formatted as Eq. 6 is utilized as the kernel function in SVM classifier, which supposes the samples subordinating to Gaussian distribution in the real world.

(6) $$K_{r\,b\,f}=\exp\left(-\gamma \cdot {||x_{1}-x_{2}||}^{2}\right).$$

where the gamma parameter γ can dominate Gaussian influence range of the support vectors in the extended space. The larger (smaller) the gamma is, the smaller (larger) the difference between samples is in the extended space, and thus an overfitting (under fitting) situation would be more likely. As for the assignment of values for the parameters {C, gamma} of SVM, we set a value interval for each of the parameters, and we adopt the GridSearch method to search the optimal values via 10-fold cross-validation. The details are given in section “Training of the SVM Classifier.”

#### Extraction of Multiple Features

To detect microbe composition in a sample, we extract three features (Coverage, GapScore, HVRScore) for each reference in the library, which are fed into the SVM classifier to predict whether this species exists in the sample.

“Coverage” is the proportion of base positions aligned by reads for all reference positions, which is positively correlated with sequencing depth. For example, if coverage of one reference is 98%, then the remaining 2% of bases are assumed to be not aligned by any reads. In the specific sequencing depth, the higher one species coverage is, the more likely it exist in the samples. We calculate the coverage of each species aligned by reads after the filtering process:

(7) $$\mathrm{Coverage}=\frac{\mathrm{the}\,\mathrm{amount}\,\mathrm{of}\,\mathrm{base}\,\mathrm{position}\,\mathrm{aligned}\,\mathrm{by}\,\mathrm{reads}}{\mathrm{the}\,\mathrm{total}\,\mathrm{length}\,\mathrm{of}\,\mathrm{reference}}$$

“GapScore” is used to measure the status of the gap for one reference after the alignment process, which can describe the existing probability of this species in the aspect of spatial distribution. The gap is a certain segment in one reference that is not mapped with any reads. In theory, for the ideal sequencing condition, we assume that one species exists in the samples if its reference is evenly covered by reads without any more gaps. However, it is a fact of unavoidable sequencing error and insufficient sequencing depth that product gaps will occur during the alignment process. The width, amount, and occurrence area (in HVRs or not) of the gap is the critical factor to identify species composition in samples. We designate G = {*g*_1_, *g*_2_, …, *g*_*n*_} as the gap set of one reference, where *g*_*i*_ is the length of the *i*-th gap. The GapScore is calculated by the following equation:

(8) $$G\,a\,p\,s\,c\,o\,r\,e=\frac{\sum_{i=1}^{n}g_{i}}{\left|G\right|}.$$

During extracting the HVRs of 16S rRNA using EMBOSS software, the V1 and V9 regions are not concluded. For V1, using the known primer pairs, only up to 25% sequences could be extracted from the total sequences; for HVR V9, the primer pairs are not found (primer pairs seen in Supplementary Table 1). Due to the high similarity of 16S rRNA gene sequences, some specific regions of one 16S reference, such as conserved regions, would be mistakenly aligned by abundant reads from other species. In this case, high variable regions, however, would be aligned by fewer reads (or even none), which generates a gap in HVRs of one reference. “HVRScore” describes the score of HVRs in one 16S, which is positively correlated with the existing probability of this reference. We assume [QSIImage] as the length of the *i*-th HVR, and *b*_*i*_ as the length of the gap in the *i*-th HVR after alignment; then, “HVRScore” of each given reference can be calculated as shown below:

(9) $$H\,V\,R\,S\,c\,o\,r\,e=\sum_{i=2}^{8}e^{-b_{i}/a_{i}}.$$

In formulation (9), using exponential operation would keep the value of the HVRScore in [0, 1]. This approach will make (Coverage, GapScore, HVRScore) three values in the same dimension.

### Abundance Estimation

Based on the predicted species using the SVM classifier, we further estimate their relative abundances. This will help to understand the abundance of each pathogenic species in a human body. Generally, the number of reads mapped to each predicted species provides an important clue for the estimation. Due to the high similarity between different species references, the phenomenon of read multiple-mapping is common. In the filtering process, the reads with small mapping scores are removed. Such a process can help to improve the prediction of species types. However, for the species abundance estimation, it is necessary to retrieve the removed multiple-mapped reads. This is because they may be originally disturbed by the non-existing species. Now, with the non-existing species discriminated from the existing species, the originally removed multiple-mapped reads might be uniquely mapped to some existing species. Based on this consideration, we re-align the multiple-mapped reads to the references of the predicted species and calculate the fractions of the species according to the number of mapped reads. For one predicted species, its abundance is estimated as the ratio of the number of mapped reads to the total number of sequencing reads.

## Results and Discussion

### Simulation Data From the 16S rRNA

To evaluate the ability of PGMicroD to detect microbe compositions and estimate the species abundances, we simulated sequence samples on the 16S rRNA in the following way. First, a vector of abundances corresponding to attained species sequences was generated by drawing from a Dirichlet distribution. Then, we use the ART (Huang et al., 2012) software to produce synthetic NGS reads according to those references. Here, the references are the sequences from the 16S rRNA. ART generates simulated sequencing reads by emulating the sequencing process with built-in, technology-specific read error models and base quality value profiles, which are parameterized empirically in a large training dataset. Hence, three types of common sequencing errors—base substitutions, insertion, and deletion—could be introduced into the simulated samples. As for the settings of sequencing read length, coverage depth, and indel error, we will make a detailed description following.

### Performance Evaluation Approach

Considering that our proposed method aims to detect types of species and their relative abundance, we choose two commonly used measurements (Sohn et al., 2014) to quantify performance: F1_score and relative root mean squared error (RRMSE), which are given as Eqs 10 and 11.

(10) $$F\,1\,\_\,s\,c\,o\,r\,e=\frac{2\cdot p\,r\,e\,c\,i\,s\,i\,o\,n\cdot r\,e\,c\,a\,l\,l}{p\,r\,e\,c\,i\,s\,i\,o\,n+r\,e\,c\,a\,l\,l}.$$

(11) $$R\,R\,M\,S\,E=\sqrt{\frac{1}{N}\cdot \sum_{i=1}^{N}\frac{{\left(\tau_{i}-t_{i}\right)}^{2}}{t_{i}}}.$$

Equation 10 is used to evaluate the performance of detecting species, where precision is the proportion of true positives in the total predicted species, and recall is the proportion of true positives in the total ground truth. In Eq. 11, *N* is the number of the true positives (truly existing species) in a sample, *t*_*i*_ is the estimated abundance of the *i*-th reference, and *τ_*i*_* is the true abundance of the *i*-th reference.

For evaluating the performance of methods on real sequencing samples, where the answers are usually unknown, we utilize the overlapping density score (ODS; Yuan et al., 2018) to evaluate performance. The ODS value of the *i*-th method can be calculated using Eq. 12.

$$O\,D\,S\,\left(i\right)=m\,e\,a\,n\,{\left(i\right)}_{o\,v\,e\,r\,l\,a\,p}\times \frac{m\,e\,a\,n\,{\left(i\right)}_{o\,v\,e\,r\,l\,a\,p}}{N\,\left(i\right)},$$

(12) $$m\,e\,a\,n\,{\left(i\right)}_{o\,v\,e\,r\,l\,a\,p}=\frac{\sum_{j=1,i\neq j}^{m}\left|S_{i}\cap S_{j}\right|}{m-1}.$$

*N*(*i*) denotes the number of species detected by the *i*-th method in one sample, *S*_*i*_ represents the set of microbial species detected by the *i*-th method, *m* is the total number of methods to be compared, and the item *mean*(*i*)_*overlap*_ denotes the mean number of species of the *i*-th method overlapping with other methods.

### Simulation Experiments on Filtering Threshold Values

Most reads from a sample suffer uncertainty in mapping to references, due to the high similarity among species and the contamination of unknown organisms. To preserve the accuracy of estimating microbial abundance in the presence of reads from unknown organisms, we implement a filter on the mapping score.

We simulated 11 samples including 1,210,410 sequencing reads with completely different composition and abundance. Specifically, we randomly select 20 references to form the original reference library as a new reference library for each sample. Then, 10 references are chosen from the new reference library as the ground-truth species for one sample. The unknown noised species, i.e., they are not included in the new reference library, are combined with ground-truth references to simulate the 16S samples. Also, the ratio of unknown noised species is set from 0.0 to 0.7 in the 11 samples. Here, the sequencing read length, sequencing error rate, and coverage depth are set to 100 bp, 0.01, and 200x, respectively.

**FIGURE 3:**
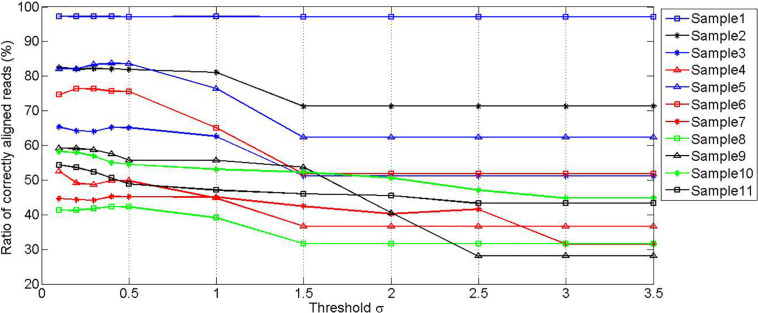
Simulation experiments showing the relationship between the ratio of correctly aligned reads and the value of threshold δ.

To find a moderate threshold value δ, a set of experiments are carried out by setting various threshold values, to observe the effect of the values on the ratio of correctly mapped reads to the unfiltered reads. The experimental results are depicted in Figure 3. It can be seen that the ratios of correctly aligned reads are at stable levels when the threshold value is less than 0.5 for all samples, and the ratios decrease gradually as the threshold value increases. It should be noted that the ratio value in the first sample is always at a constant level. This is because this sample is not polluted by any noise species. To reasonably filter the potentially mistakenly mapped reads, we choose 0.44 as the mapping score threshold δ.

### Training of the SVM Classifier

**FIGURE 4:**
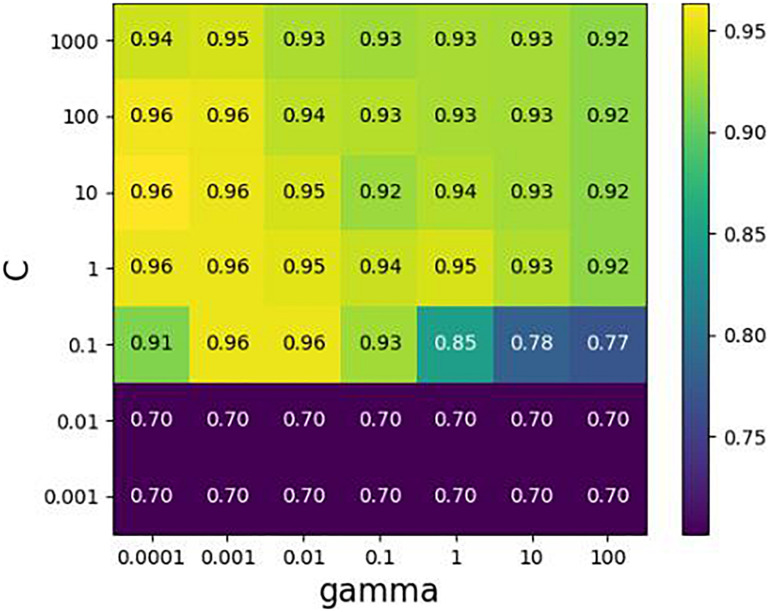
Cross-validation with GridSearch about parameters {C, gamma} in SVM. The horizontal axis represents the changing of gamma, the vertical axis the changing of C, and value in the grid indicates an average F1-score of cross-validation.

In cooperation with Diagnostics Inc., we constructed a species library of 150 common clinical microorganisms. These microorganisms include Staphylococcus, Streptococcus, Pseudomonas aeruginosa, Proteus, Salmonella, and other rare pathogens. They are distributed in 9 families, 18 classes, 35 orders, 52 families, and 86 species. It is noted that some strains are at the subspecies level. We extracted the features of simulated samples to train the SVM classifier (Chauhan et al., 2018), where the true species are known. Each species in a sample is formatted by a vector with four elements (Label, Coverage, GapScore, and HVRScore). Here, the first element “Label” is assigned with “1” or “0,” representing if the corresponding species exists or not. We align 11 simulated samples (designed in section “Simulation Experiments on Filtering Threshold Values”) to our species library including 150 clinical pathogenic microbes. We collect 1650 species vectors including 110 positive and 1540 negative vectors, and then randomly divide them into two groups according to the ratio of 3:1. The first group is a training dataset, which is used to make 10-fold cross-validation to search the optimal parameters {C, gamma} of SVM by GridSearch, and the second group is test dataset, which is used to test the generalization performance of SVM. As shown in Figure 4, when the parameter values of C and gamma are set to 0.1 and 0.01, respectively, the average F1_score gets the best value 0.957 for the validation dataset. The F1_score could achieve up to 0.917 for the testing dataset. As such, the accuracy of detecting species in the validation dataset is similar to that in the testing dataset. Hence, we believe that the proposed method possesses strong generalization ability and is expected to be a useful tool for the detection of species in new samples.

### Analysis of the Effect of Coverage Depth on the Performance

**FIGURE 5:**
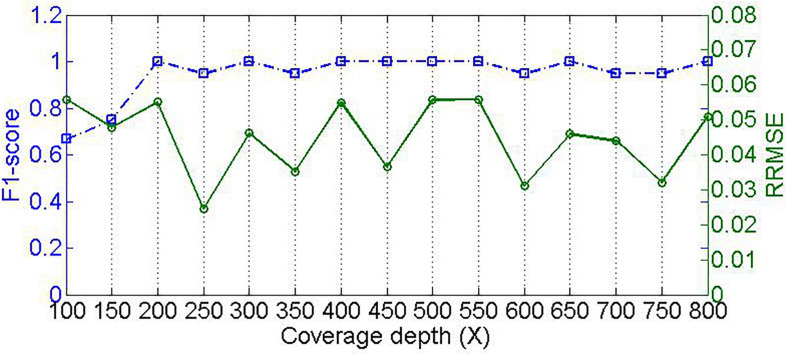
Effect of sequencing coverage depth on PGMicroD. The horizontal axis is the value of coverage depth, the left vertical axis represents F1-score, and the right vertical axis is the RRMSE.

Different samples are usually sequenced at different levels of coverage depths, which might pose an influence on the performance for detecting species and estimating their relative abundances. To validate this, we designed experiments to test our method by changing the sequencing coverage depth. We simulate 15 samples with read length of 100-bp, insertion and deletion rate of 0.01, at coverage depth ranging from 100 to 800×. As shown in Figure 5, we could see that the performance of composition detection increases substantially as sequencing coverage depth increases, whereas the performance of abundance estimation is slightly affected by the coverage depth. For example, in the case of coverage depth 100×, the F1_score of detecting species is around 0.67; when the coverage depth is at 200×, the F1_score is close to 1.0. This is likely because in the sample with low sequencing coverage depth, the reads are not sufficient for supporting feature values that discriminate species references. Alternatively, the mapping score of each read is not severely influenced by coverage depth. This is because the score is only related to the read alignment status rather than coverage depth, and thus the filtering of residual reads cannot be affected by coverage depth. Accordingly, the microbial abundance estimation is not significantly affected by coverage depth.

### Analysis of the Effect of Read Length on Performance

**FIGURE 6:**
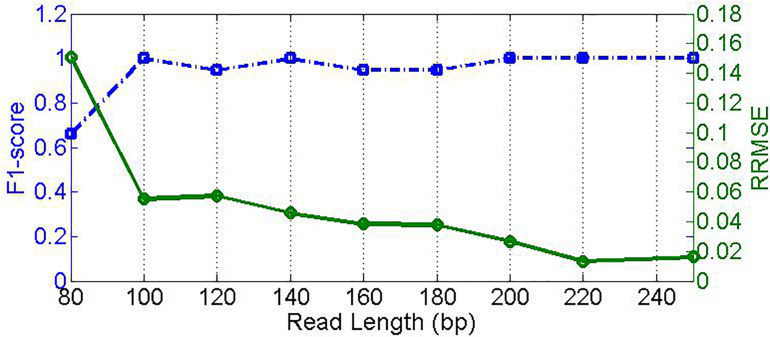
Effect of read length on PGMicroD. The horizontal axis is the value of read length, the left vertical axis represents F1-score, and the right vertical axis is RRMSE.

Read length is one of the most important characters of NGS data; different read lengths significantly influence the read alignment process. Generally, the larger the read length is, the more accurate the alignment is. An accurate alignment will provide a solid foundation for downstream analysis of the sequencing read data. Since different sequencing platforms usually produce reads with distinct scopes of read length, it is necessary to investigate the effect of the read length on the performance of our proposed method. To do this, we simulate nine samples with read length ranging from 80 to 250 bp. Here, the sequencing coverage depth is set to 500x, and both insertion and deletion error rates are set to 0.01. The experimental result is depicted in Figure 6, where the F1_score increases sharply from around 0.67 at read length of 80 bp to around 0.99 at read length of 100 bp, and the RRMSE decreases from 0.16 to below 0.06. When the read length is increasing, the F1_score is roughly at a level of 1.0, and the RRMSE further decreases to below 0.02. This suggests two implications. First, high-quality alignment due to long read lengths can enable high-quality feature values to discriminate microbial species. Second, our proposed PGMicroD method can obtain a reasonable performance without the requirement of extremely long read length. Currently, read length over 100 bp is easy to achieve with most of the existing sequencing platforms. Therefore, we conclude that PGMicroD has practical application value in the analysis of NGS sequencing data.

### Analysis of the Effect of Sequencing Error on the Performance

**FIGURE 7:**
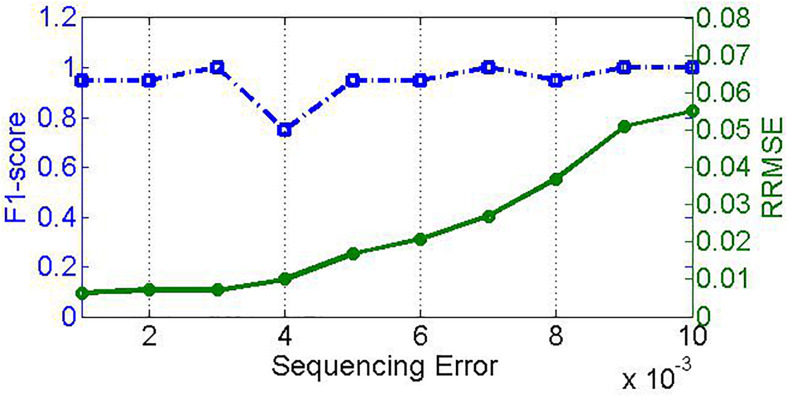
Effect of sequencing error on PGMicroD. The horizontal axis is the value of sequencing error, the left vertical axis represents F1-score, and the right vertical axis is RRMSE.

Sequencing error is another factor closely associated with NGS technology. Generally, sequencing errors include substitution error and insertion/deletion error. To investigate the effect of sequencing errors on the performance of PGMicroD, we carry out experiments on sequencing datasets with different sequencing error rates. For this, we simulate 10 samples with insertion/deletion error rate ranging from 0.001 to 0.01 and an empirical substitution error rate. Here, the sequencing coverage depth is 500× and the read length is 100 bp. The experimental result is depicted in Figure 7; the F1_score remains relatively constant whereas the RRMSE increases with an increase in sequencing error rate.

### Comparison With Other Methods on Simulation Experiments

Our proposed method considers the fact that the samples would have contamination from unknown noised organisms and human sequencing reads. To test the performance of PGMicroD against peer methods, we simulate 30 samples with a read length of 100 bp, coverage depth of 800×, and insertion/deletion error rate of 0.01. In these samples, we consider the noise reads originated from non-ground-truth species (other organisms) and human genomes (simultaneously). We define *f*_1_ as the abundance of noised reads from non-ground-truth species, *f*_2_ represents the abundance of noised reads from human genomes, and *f*_3_ represents the abundance of ground truth species. Here, the summation of the three items, *f*_1_ + *f*_2_ + *f*_3_, equals to 1.

When simulating samples, we set *f*_2_ varying from 0.0 to 0.8 step at 0.2. First, we choose one specific value as *f*_2_, then divide the value (1-*f*_2_) into *f*_1_ and *f*_3_, which are satisfied for *f*_1_:*f*_3_ = 0.0:1.0, 0.16:0.84, 0.285:0.715, 0.375:0.625, 0.44:0.56, and 0.5:0.5. In this way, we can obtain 30 simulated samples. We perform PGMicroD and five other methods and compare their results in terms of F1_score and RRMSE, including Karp, Harp, Kallisto, Bwa, and Mothur, as shown in the Supplementary Figures 1–4. Supplementary Figures 1, 3 show the changes of F1_score and RRMSE with the changes of *f*_1_, wherein in each subfigure, the value of *f*_2_ is constant. We note that the performance of all six methods tends to decrease with *f*_1_ increasing. This is because non-ground-truth species are very similar to the ground-truth species, posing influence on read alignment. Supplementary Figures 2, 4 show the changes of F1_score and RRMSE with the changes of *f*_2_. In each subfigure of these figures, the ratio between *f*_1_ and *f*_3_ is constant. We note that the F1_score of most of the six methods are relatively unchanging when *f*_2_ is increasing, and the RRMSE values of the Harp and Mothur methods are increasing while the other four methods tend to decrease. Comparatively, the influence of noise from human genomes is less than the influence of noise from non-ground-truth species.

In these comparative experiments, PGMicroD achieves the highest F1_score in most situations of the simulation configurations, followed by Karp, Harp, Kallisto, Bwa, and Mothur. In terms of RRMSE, Kallisto performs the best, followed by PGMicroD, Bwa, Harp, Karp, and Mothur. We may conclude that PGMicroD obtained the best trade-off between F1_score and RRMSE, followed by Harp and then other methods.

### Comparison With Peer Methods on Real Sequencing Samples

**FIGURE 8:**
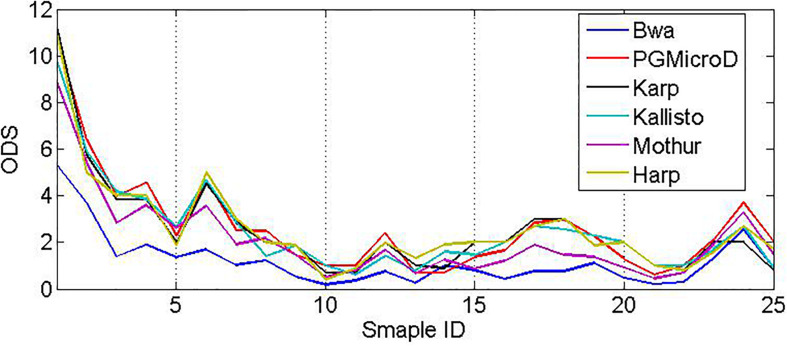
Performance comparison among the six methods based on real samples based on ODS scores.

Every real sample is composed of more than 120,000 sequencing reads that are collected from urine, cerebrospinal fluid, or blood by using the Ion Torrent sequencing platform. The average length of the reads is around 113 bp, and the sequencing coverage depth is around 1000x. First, we use the primer pairs to extract HVRs of each species in the library. Then, we align the real samples (fastq file) to the species library and compute the mapping score of every read according to its sequencing quality, mapping gaps, and status of following to HVRs. We filter the sequencing reads with a low score, and we calculate three signals (Coverage, GapScore, and HVRScore) of each species in the library. Next, we feed those vectors into the SVM classifier to determine which species exist in the sample. Also, we apply Karp, Harp, Kallisto, Bwa, and Mothur to these samples for microbial composition detection. The comparative result is depicted in Figure 8. The average ODS value of PGMicroD, Bwa, Karp, Harp, Kallisto, and Mothur at 25 samples are 2.28, 0.68, 2.16, 1.96, 2.2, and 1.52, respectively. Also, we plot Venn diagrams to show the overlapped species detected by the six methods for each sample in Supplementary Figure 5. From these experiments and comparisons, we conclude that PGMicroD performs best among the six methods. Our proposed method is very suitable for the analysis of samples where read length is more than 100 bp, coverage depth is more than 200x, and the sequencing error rate is lower than 0.01.

## Discussion and Conclusion

In the above experiments, we note that the noised unknown species exert a negative influence on composition detection. This is because noised species are very similar to the ground truth species; in such a situation, the reads coming from the noised species are aligned well to the reference database. Also, the reads from noised species will increase the estimated abundance of the ground truth species. However, this negative effect can be minimized by implementing a mapping score filter, which can remove most of the reads originating from unrelated species. As for noised human sequencing reads, their influence is less than the influence of noised species on composition detection and abundance estimation. This is because the sequences of human genomes are rather different from the focal species. Harp displays the best performance on composition detection in samples without noised unknown species and human sequencing reads, but the deviation of the abundance estimation is not highly significant. This is because Harp assumes that all reads in the samples originated from species present in the reference database. The operating time of Mothur is longest among the methods, and it occupies a large amount of memory while running because the distance between any two reads is computed and then utilized as an OTU cluster analysis. The false-positive rate of compositions detected by BWA is high, meanwhile, and it cannot judge whether the species with low abundance exist in the sample or not. We found the same pattern for Kallisto. Karp provides an impressive abundance estimation result via re-estimating the abundance of species detected by Kallisto, but the performance of composition detection is not as significant as PGMicroD. In a series of samples with different noised species, the performance of PGMicroD is higher compared to the other methods, due to the HVRs and spatial distribution characteristics. However, the efficiency of PGMicroD is second to Karp because of the time to calculate the mapping score of all the reads. On the whole, among the six methods, our proposed method performs better than the other five.

A key to microbial research is to detect composition and estimate abundance with high performance. In this paper, we propose a new algorithm, PGMicroD, for the detection of pathogenic microbial composition using NGS data. It can be easily executed via a command line described in the software package.^1^ The most important feature of PGMicroD is that it extracts multiple features to train a classifier to predict species and performs a second-round alignment to estimate abundances. Generally, each of the selected features (Coverage, GapScore, and HVRScore) may have its own marginal effect and may have different effects on the microbial composition prediction. According to our observation on experiments, GapScore poses larger effect on the detection of the microbial composition than the other two features. We test PGMicroD based on both simulation and real datasets, indicating the PGMicroD exhibits superior performance. In future work, we intend to integrate mutations such as single nucleotide variations (Yuan et al., 2020) and copy number variations (Xi et al., 2019; Zhao et al., 2020) to improve the detection of microbial composition. We also plan to establish a more comprehensive reference library for detecting species and improving detection accuracy and create new methods aiming at the filtered reads to identify new species.

## Data Availability Statement

The datasets presented in this study can be found in online repositories. The names of the repository/repositories and accession number(s) can be found below: PGMicroD [https://github.com/BDanalysis/PGMicroD].

## Author Contributions

HZ and SW participated in the design of algorithms and experiments. SW participated in the design of the whole framework of detecting CNVs and participated in the analysis of the performance of the proposed method, XY directed the whole work and conceived of the study and help edit the manuscript. All authors read the final manuscript and agreed on the submission.

## Conflict of Interest

The authors declare that the research was conducted in the absence of any commercial or financial relationships that could be construed as a potential conflict of interest.

## Footnotes

1 https://github.com/BDanalysis/PGMicroD
