# Supplementary material for: Detection of Pathogenic Microbe Composition Using Next-Generation Sequencing Data
Source: Front Genet. 2020 Nov 30;11:603093. doi: 10.3389/fgene.2020.603093 (PMC7734255; doi:10.3389/fgene.2020.603093)
Supplement: Supplementary file 1 [file Data_Sheet_1.docx]

Supplementary Material

# The primer pairs list

**Supplementary Table 1.** The primer pairs list to extract HVRs of 16S

| **No.** | **forward primer sequence** | **Backward primer sequence** | **HVR** |
| --- | --- | --- | --- |
| 1 | AGYGGCGNACGGGTGAGTAA | TGCTGCCTCCCGTAGGAGT | V2 |
| 2 | CCTACGGGAGGCAGCAG | ATTACCGCGGCTGCTGG | V3 |
| 3 | AYTGGGYDTAAAGNG | TACNVGGGTATCTAATCC | V4 |
| 4 | AGGATTAGATACCCT | CCGTCAATTCCTTTGAGTTT | V5 |
| 5 | TCGAtGCAACGCGAAGAA | ACATtTCACaACACGAGCTGACGA | V6 |
| 6 | GYAACGAGCGCAACCC | GTAGCRCGTGTGTMGCCC | V7 |
| 7 | ATGGCTGTCGTCAGCT | ACGGGCGGTGTGTAC | V8 |

# Result on simulation data

In thirty simulated samples, we consider the noise reads originated from non-ground-truth microbes and human genomes simultaneously. We define that *f*_1_ represents the abundance of noised reads from non-ground-truth microbes, *f*_2_ represents the abundance of noised reads from human genomes, and *f*_3_ represents the abundance of ground truth species. Here, the summation of the three items, *f*_1_+*f*_2_+*f*_3_, equals to 1. During simulating samples, we set *f*_2_ varying from 0.0 to 0.8 step at 0.2. Fist, we choose one specific value as *f*_2_, then divide the value (1-*f*_2_) into *f*_1_ and *f*_3_, which are satisfied of *f*_1_:*f*_3_ = 0.0:1.0, 0.16:0.84, 0.285:0.715, 0.375:0.625, 0.44:0.56, 0.5:0.5. In this way, we can obtain 30 simulated samples. We perform PGMicroD and other five methods and compare their results in terms of F1-score and RRMSE, as shown in Figure 1 to Figure 4. Figure 1 and Figure 3 show the changes of F1-score and RRMSE along with the changes of *f*_1_, where in each subfigure, the value of *f*_2_ is constant. We can note that the performance of all the six methods tends to decrease when *f*_1_ is increasing. This can be explained by that non-ground-truth microbes are very similar to the ground truth species, posing a great influence on read alignment. Figure 2 and Figure 4 show the changes of F1-score and RRMSE along with the changes of *f*_2_, where in each subfigure, the ratio between *f*_1_ and *f*_3_ is constant. We can note that the F1-scores of most of the six methods are almost not changed when *f*_2_ is increasing, and the RRMSE values of the Harp and Mothur methods are increasing while the other four methods tend to decreasing. Comparatively, the influence of noise from human genomes is less than the influence of noise from non-ground-truth microbes.


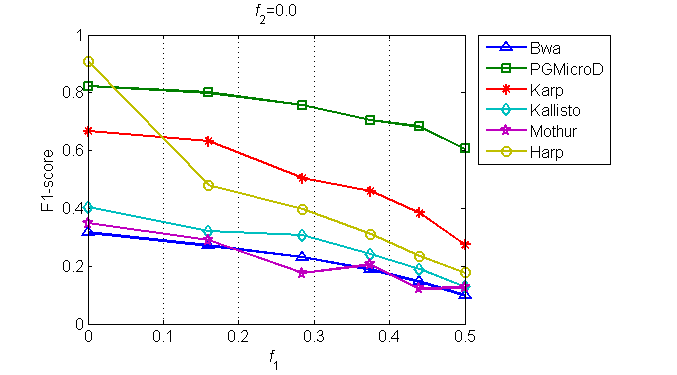

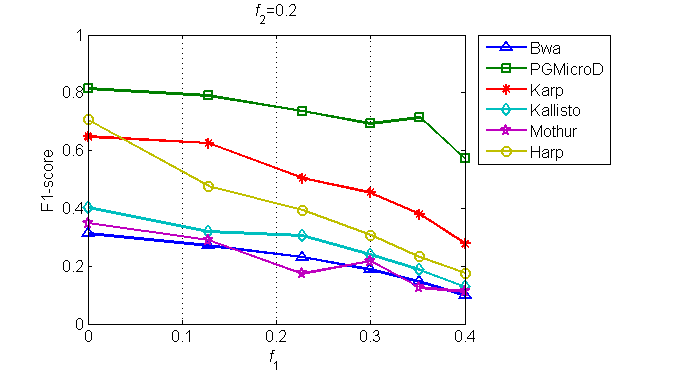

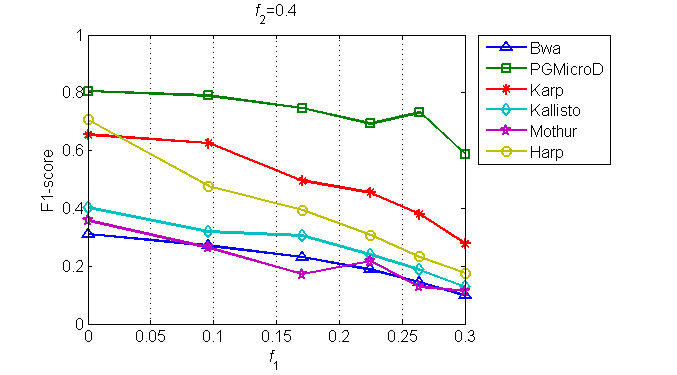

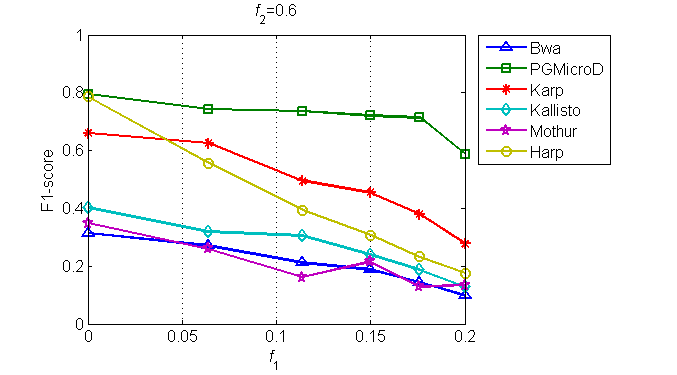

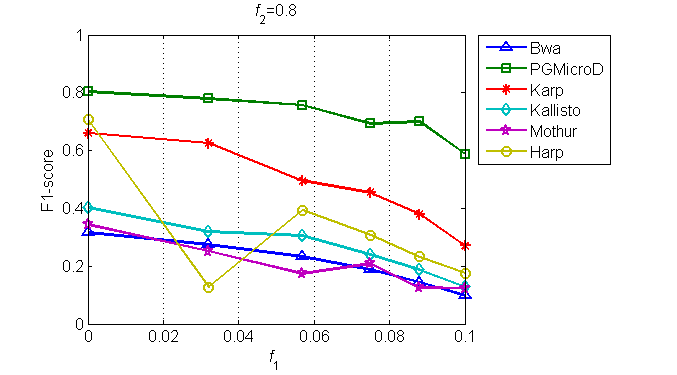


**Supplementary Figure 1.** Comparison of F1-socre between the six tools. The horizontal axis in each subfigure denotes the noise from non-ground truth species at a fraction falling in a specific interval.


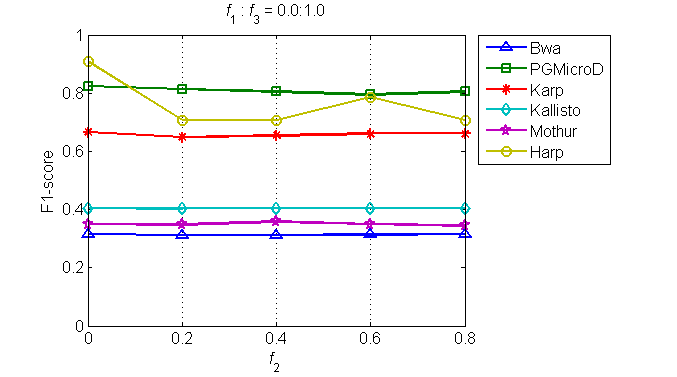

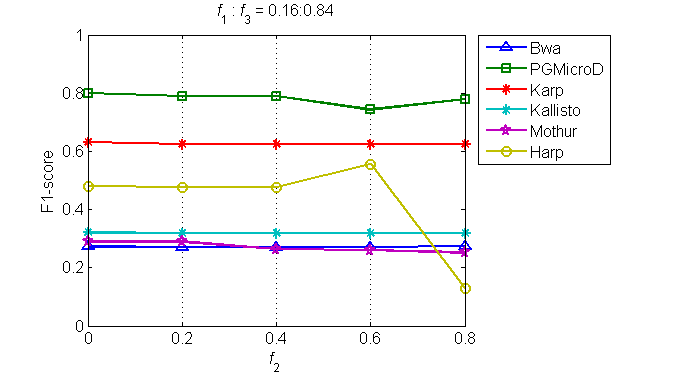

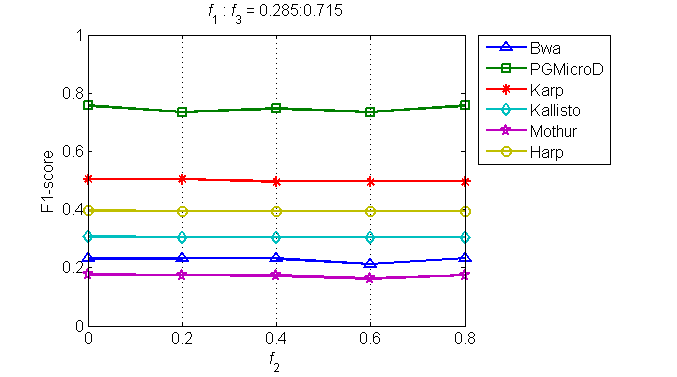

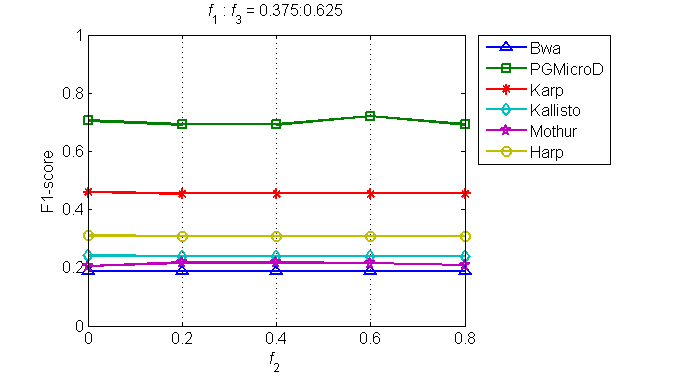

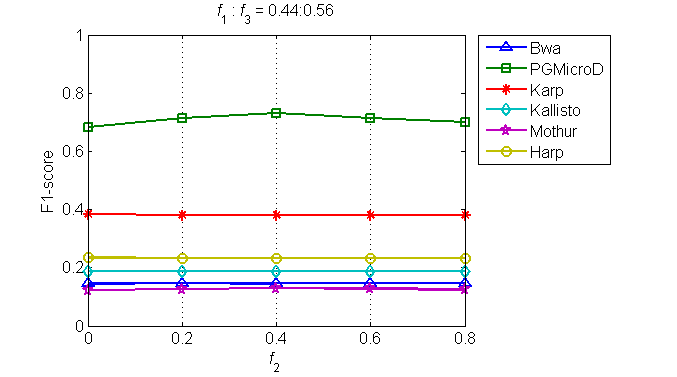

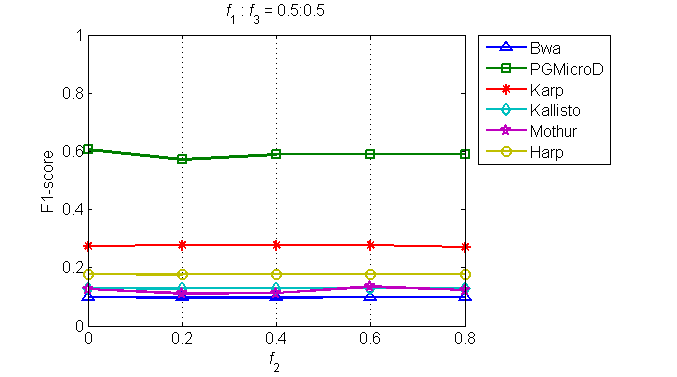


**Supplementary Figure 2.** Comparison of F1-socre between the six tools. The horizontal axis denotes the noise from human genomes at a fraction ranging from 0.0 to 0.8.


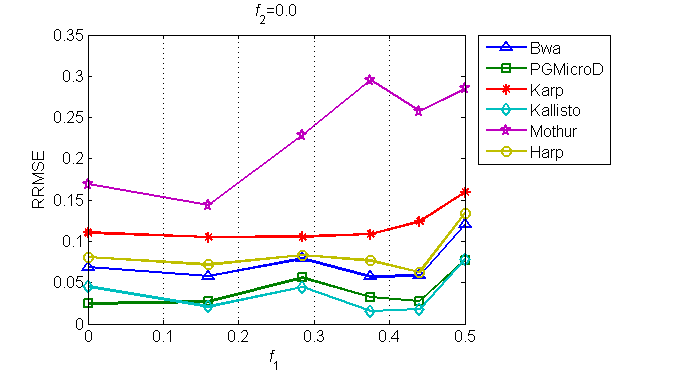

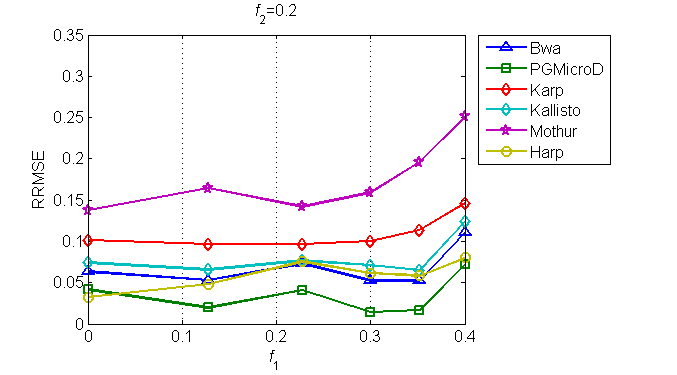


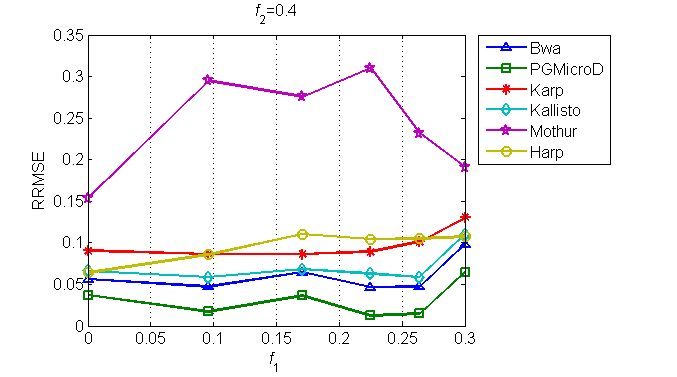

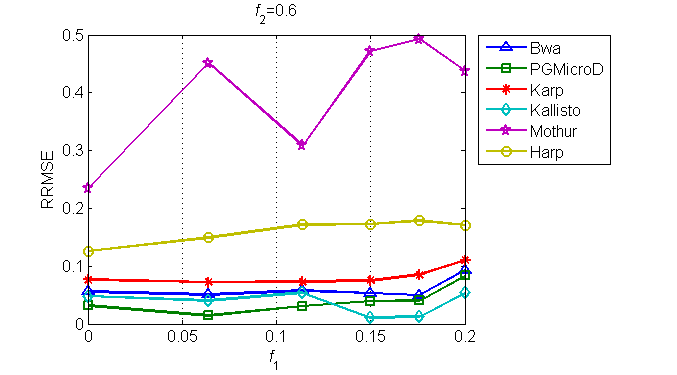


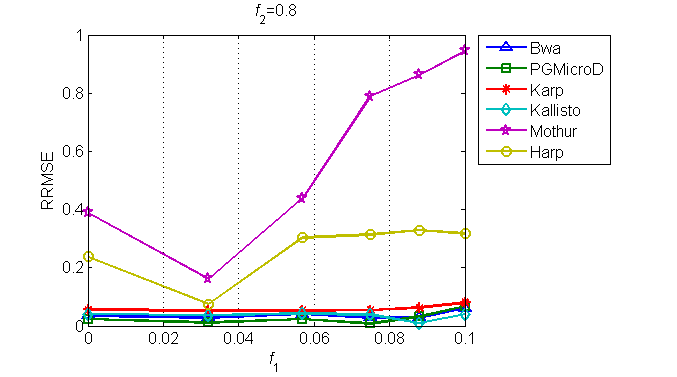


**Supplementary Figure 3.** Comparison of RRMSE between the six tools. The horizontal axis in each subfigure denotes the noise from non-ground truth species at a fraction falling in a specific interval.


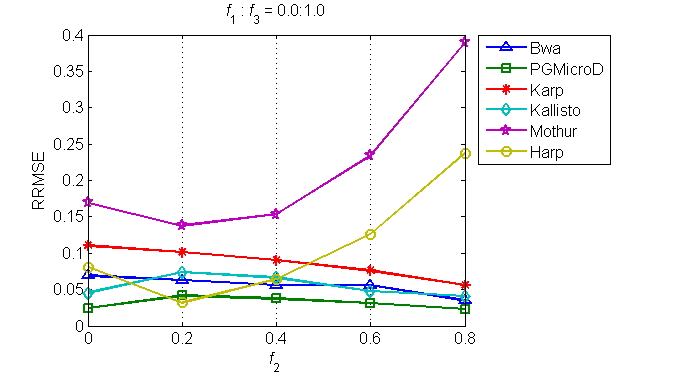

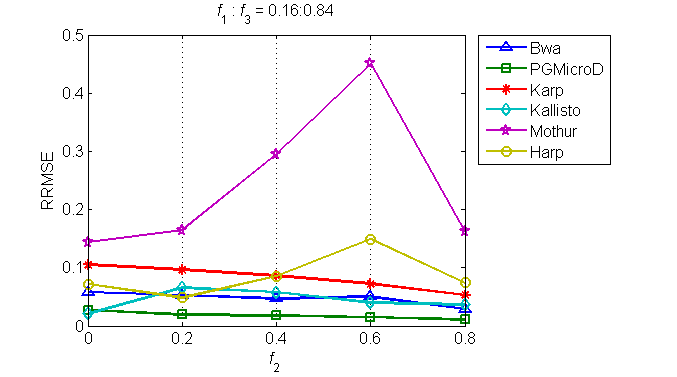

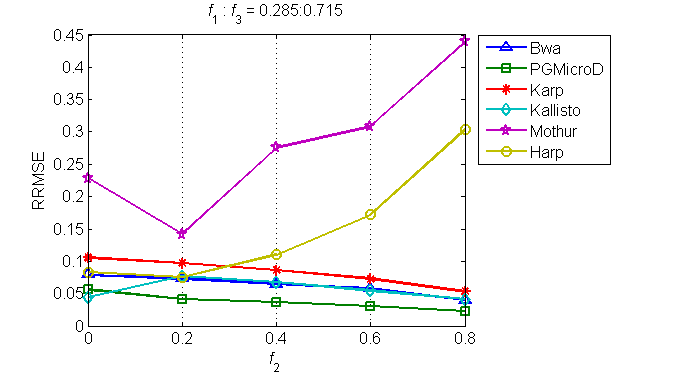

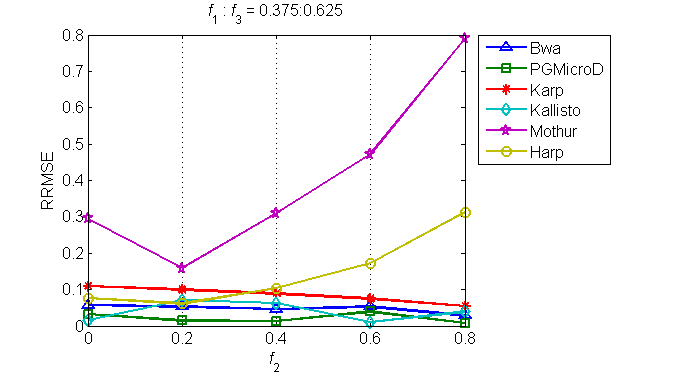

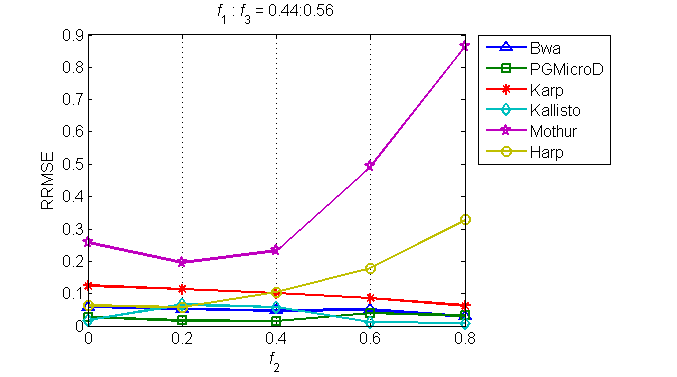

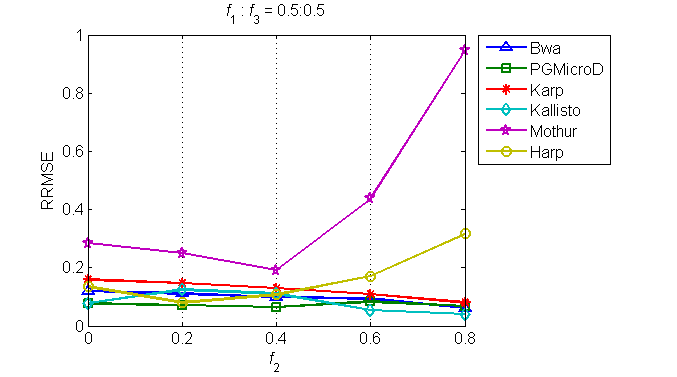


**Supplementary Figure 4.** Comparison of RRMSE between the six tools. The horizontal axis denotes the noise from human genomes at a fraction ranging from 0.0 to 0.8.

# Result on real data

The real data were collected from urine, cerebrospinal fluid or blood by using Ion Torrent sequencing platform. The average length of the reads is around 113 bp and the sequencing coverage depth is around 1000x. We apply PGMicroD, Karp, Harp, Kallisto, Bwa and Mothur to these samples for microbial composition detection. We plot Venn diagrams to show the overlapped species detected by the six methods for each sample in Figure 5.

1)
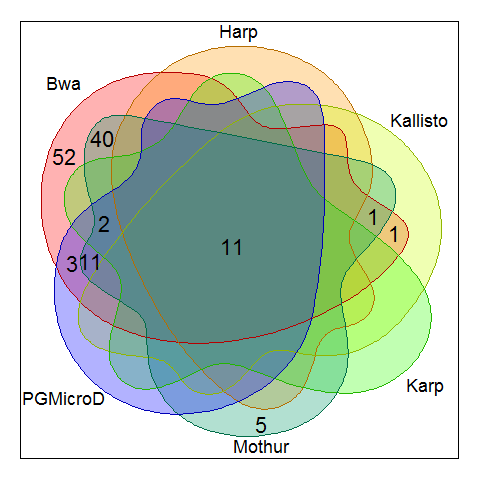
2)
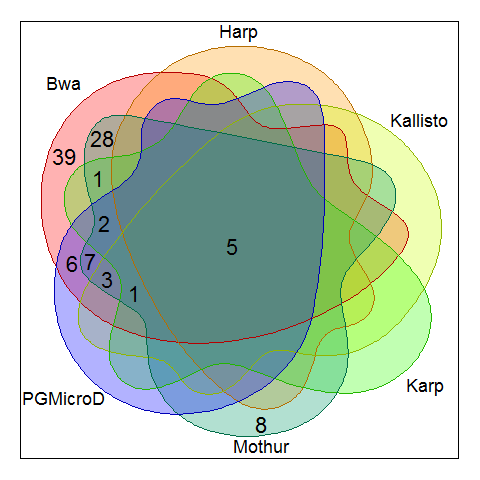
3)
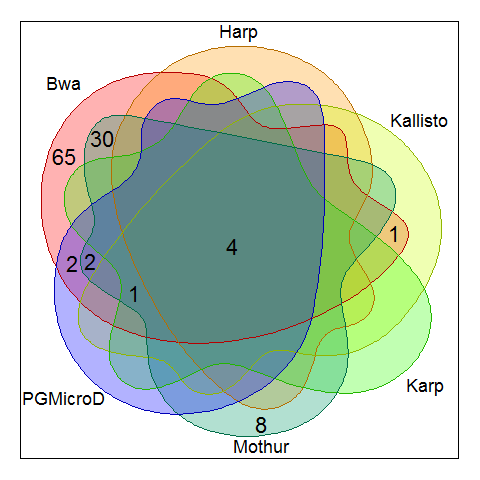


4)
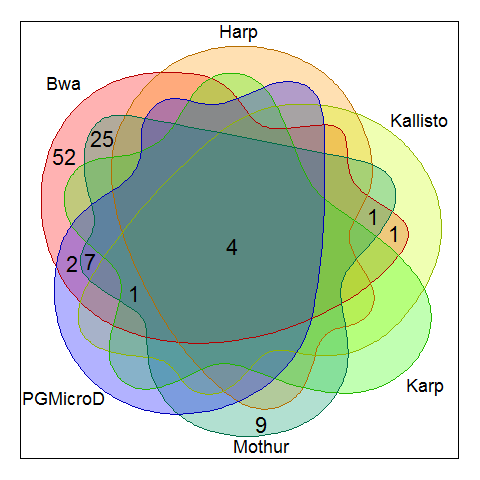
5)
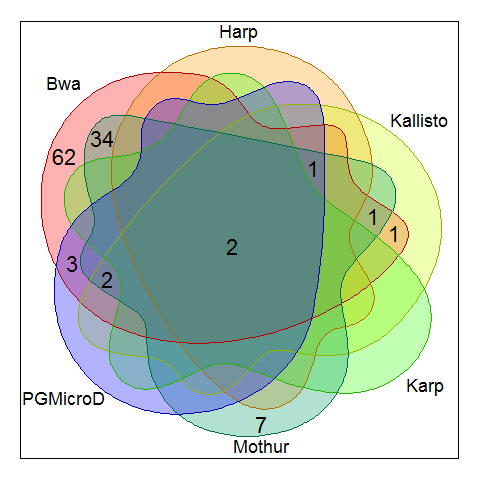
6)
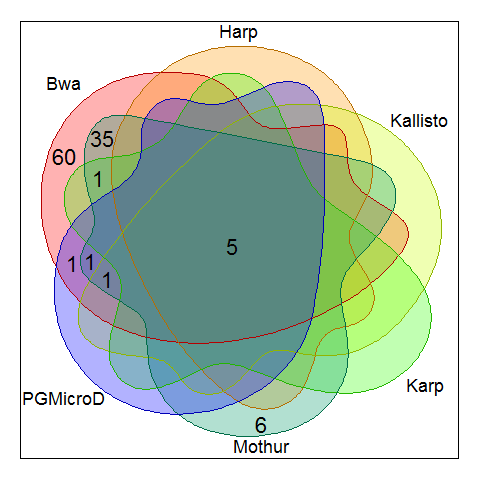


7)
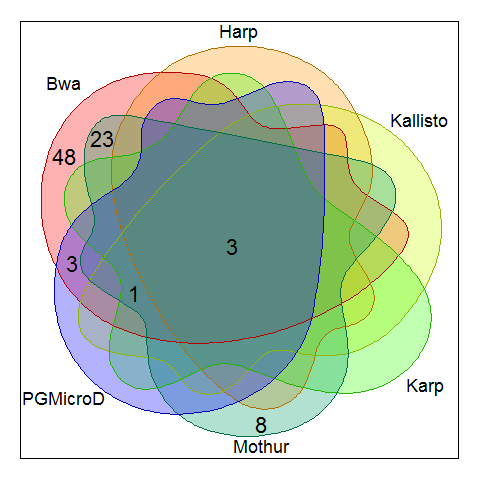
8)
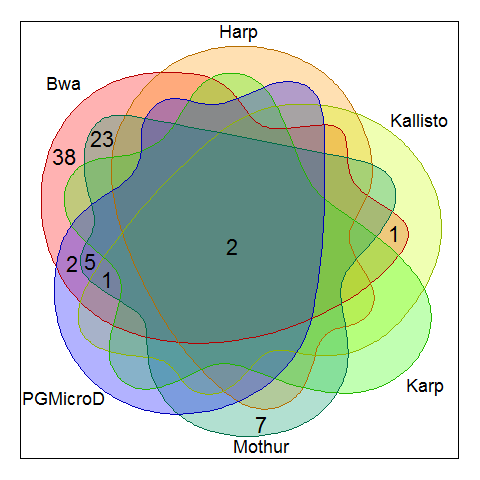
9)
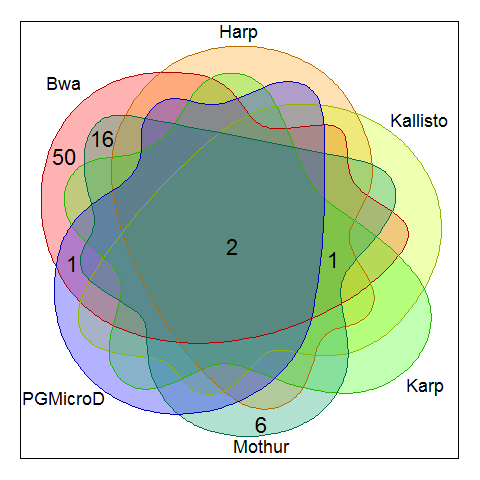


10)
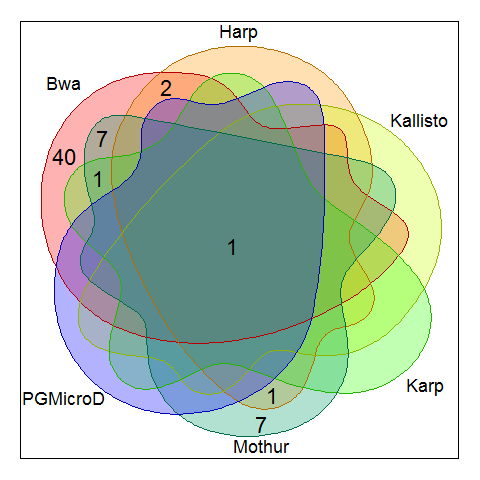
11)
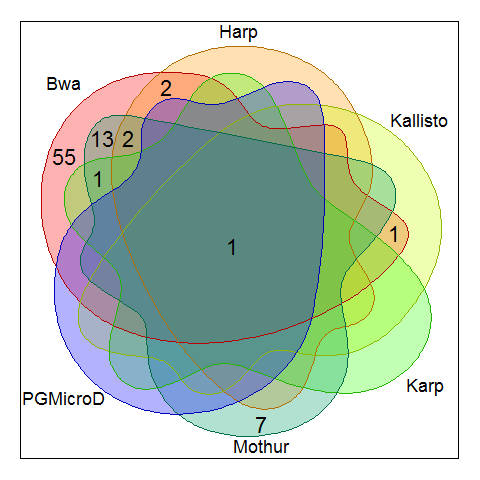
12)
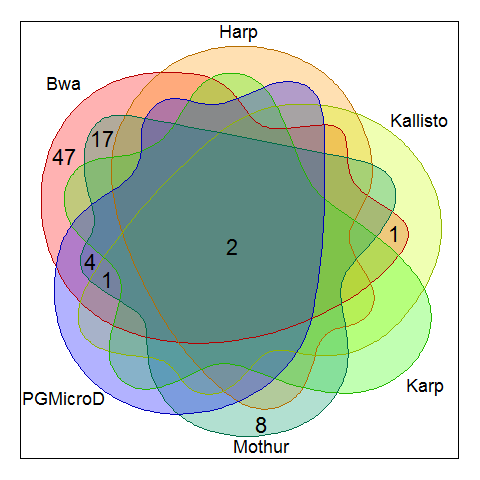


13)
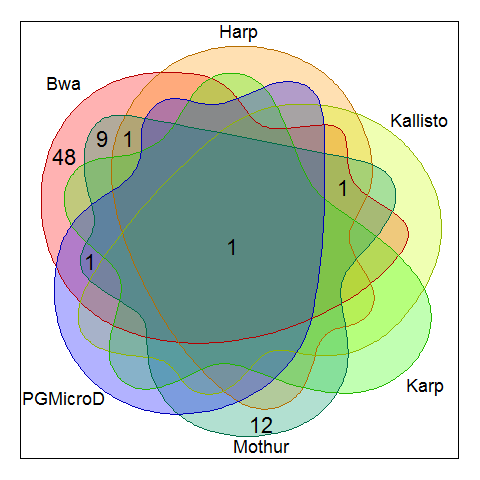
14)
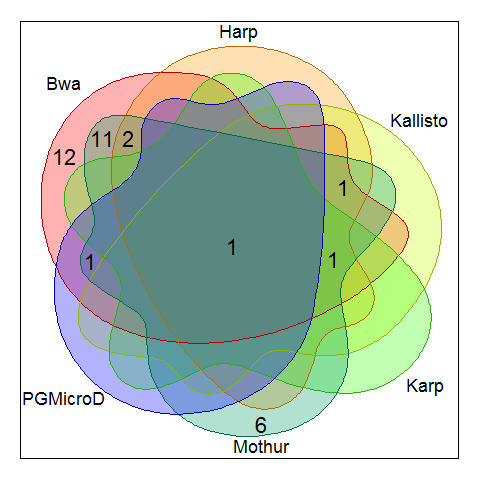
15)
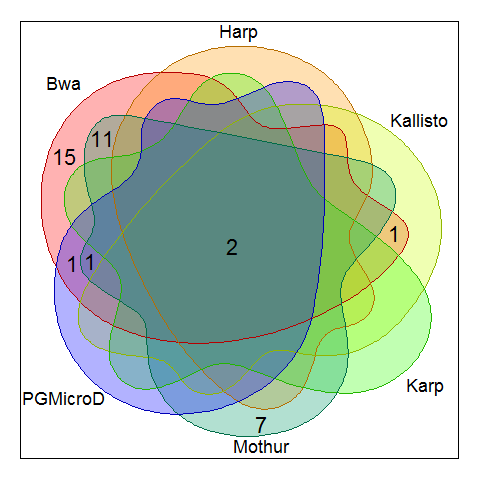


16)
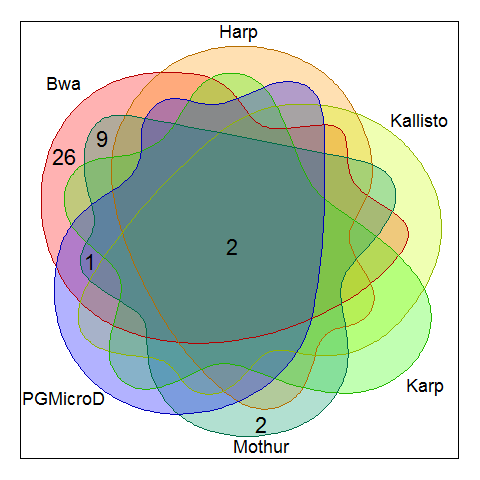
17)
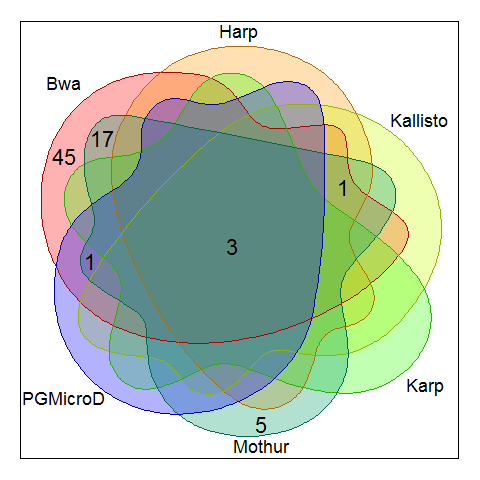
18)
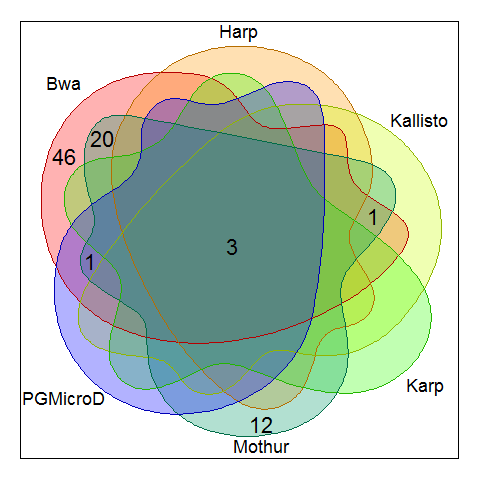


19)
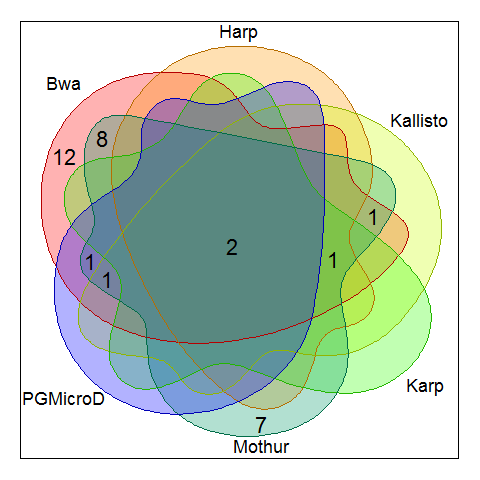
20)
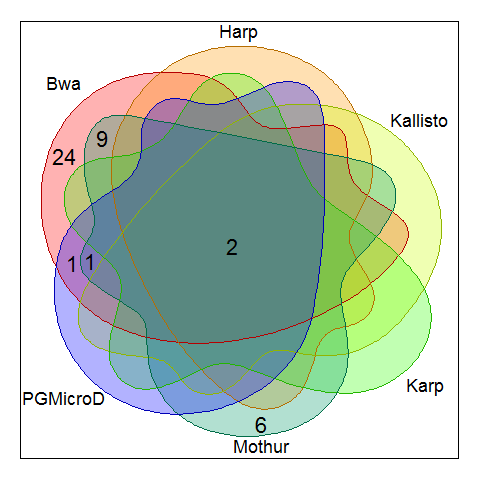
21)
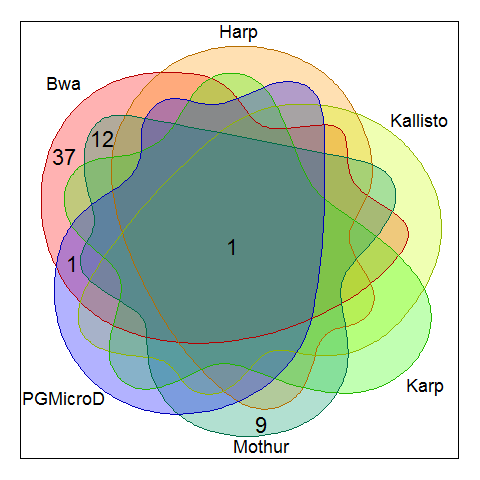


22)
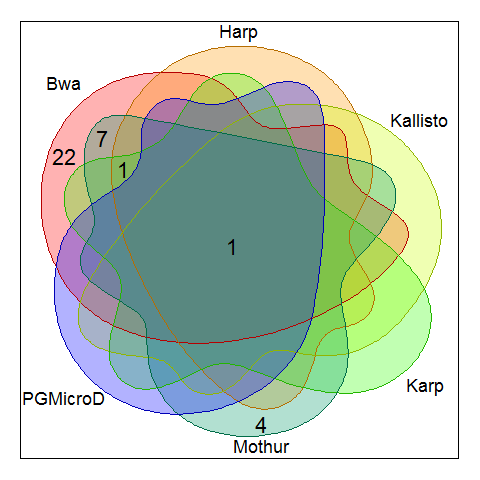
23)
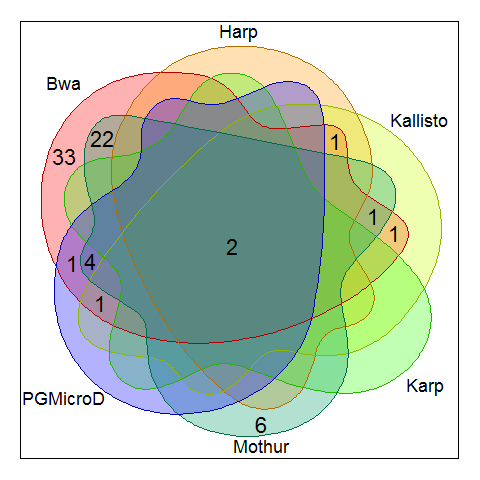
24)
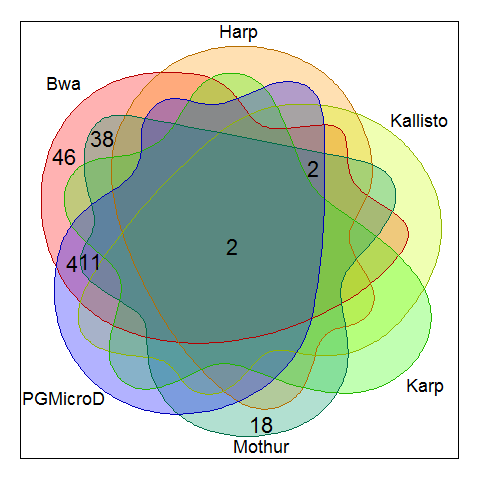


25)
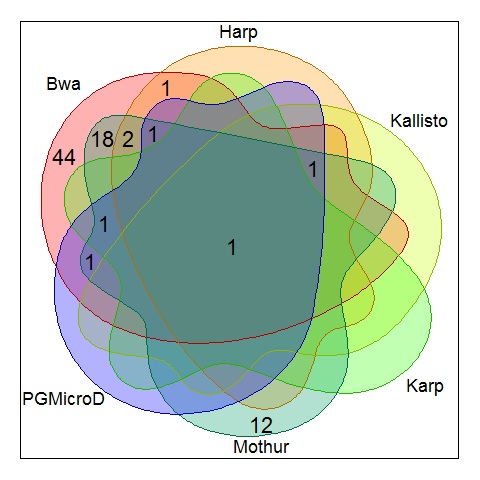


**Supplementary Figure 5.** Venn diagrams to show the overlapped species detected by the six methods for each sample.
